# Concomitant renal cell carcinoma and chronic myelogenous leukemia: use of a targeted approach

**DOI:** 10.3747/co.v16i2.301

**Published:** 2009-03

**Authors:** S.K. Pal, R.K. Gupta, G. Dosik, R.A. Figlin

**Affiliations:** * City of Hope Comprehensive Cancer Center, Duarte, CA, U.S.A

**Keywords:** Imatinib, bevacizumab, renal cell carcinoma, chronic myelogenous leukemia, erlotinib

## Abstract

Numerous therapeutic options have been introduced for metastatic renal cell carcinoma (mrcc) in recent years, including monoclonal antibodies such as bevacizumab and small-molecule tyrosine kinase inhibitors such as sunitinib and sorafenib. Similarly, several other small-molecule inhibitors—including imatinib, dasatinib, and nilotinib—have been approved for the treatment of chronic myelogenous leukemia (cml). The combination of these targeted agents is an area of intense clinical investigation. Here, we describe a patient diagnosed with mrcc while on imatinib therapy for cml. Treatment of this patient with the combination of bevacizumab and imatinib led to a 6-month period of stable disease, with no treatment-related adverse events. More extensive clinical exploration of this combination of agents may therefore be warranted.

## 1. CASE PRESENTATION

Upon abdominal computed tomography (ct) imaging performed to rule out nephrolithiasis, a 57-year-old woman was incidentally found to have a 5-cm renal mass. The patient subsequently underwent a right-sided nephrectomy, and pathology analysis of the surgical specimen revealed a 5-cm Fuhrman grade 2 clear-cell-type renal cell carcinoma. Imaging by ct of the chest, abdomen, and pelvis at that time showed no evidence of distant disease, and the patient was followed clinically by her urologist.

Roughly 2 years later, the patient presented to her primary care practitioner with vague complaints of fatigue and lethargy. Initial workup included a complete blood count, remarkable for a pronounced leucocytosis (30,000 white cells/μL, normal differential). Subsequent bone marrow examination was consistent with a diagnosis of chronic myelogenous leukemia (cml), and the patient was initiated on therapy with imatinib. The patient tolerated therapy with minimal adverse effects, and within 3 months of therapy initiation, she had mounted a complete cytogenetic response by peripheral blood Bcr–Abl mutation screening.

The patient continued to demonstrate a durable remission on imatinib therapy. However, at a time corresponding to roughly 4 years of imatinib therapy, the patient was noted to have onset of cough and shortness of breath. Imaging of the chest by ct revealed multiple pulmonary nodules, and ct-guided biopsy of a representative lesion was consistent with clear-cell carcinoma of the kidney. After a discussion of potential therapeutic options, the patient was initiated on therapy with bevacizumab while continuing treatment with imatinib.

**FIGURE 1:**
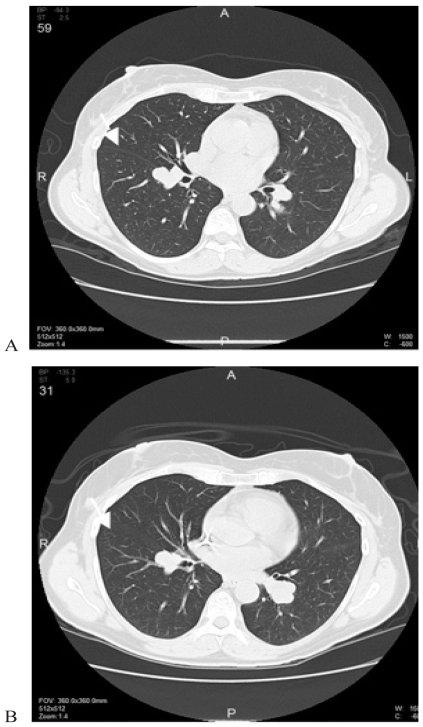
Computed tomography imaging demonstrates no appreciable change in an index lesion (arrow) in the left lung (A) at the time of initiation of therapy with bevacizumab and imatinib, and (B) 6 months after initiation of therapy.

Six months after initiation of the bevacizumab therapy, interval imaging data shows no significant change (Figure 1), and the patient has experienced no toxicities related to either therapeutic agent.

## 2. DISCUSSION

### 2.1 Targeted Therapy for mRCC and CML

Chronic myelogenous leukemia and metastatic renal cell carcinoma (mrcc) represent two disorders whose therapies have been revolutionized by the advent of targeted agents. The natural history of cml has been drastically altered by the Bcr–Abl tyrosine kinase inhibitor (tki) imatinib. Use of imatinib has been validated with 5-year follow-up data from a study of 1106 patients randomized to either imatinib or interferon alfa plus cytarabine. In that study, a complete cytogenetic response was obtained in 69% of patients by 12 months and in 87% of patients by 60 months^1^.

Although imatinib provides a durable remission, patients refractory to the drug also have therapeutic alternatives such as dasatinib or nilotinib. Dasatinib, a Bcr–Abl tki that targets most imatinib-resistant *BCR–ABL* mutations, was shown to cause a complete hematologic response in 93% of patients assessed with chronic-phase cml, and a major hematologic response in 70% of patients with accelerated-phase disease 2. A similar study to assess the activity of nilotinib also yielded data suggesting activity of this novel tki in the setting of imatinib-resistant cml^3^.

Emerging data also suggest the utility of a targeted approach to the treatment of mrcc. Based on results of a meta-analysis assessing six prospective trials, interferon alfa had previously been accepted as the standard treatment for mrcc. A total of 463 patients with mrcc had received therapy with interferon alfa, yielding a median time to progression of 4.7 months and a median overall survival (os) of 13.1 months^4^. Those data led to the use of interferon alfa as a comparator in the phase iii trial of sunitinib, an oral tki targeting the vascular endothelial growth factor receptor (vegfr) and platelet-derived growth factor receptor (pdgfr)^5^. In 750 patients with previously untreated clear-cell mrcc, an improvement in progression free survival (pfs) was observed with sunitinib [10.9 months vs. 5.1 months; hazard ratio (hr): 0.42; *p* < 0.001], paralleled by improvements in quality of life^6^.

A distinct targeted approach has been used for patients refractory to standard therapy. In a trial using the agent sorafenib (a tki with inhibitory activity against vegfr, pdgfr, Flt-3, and c-Kit) or placebo in such patients, a benefit in time to progression was observed with sorafenib (5.5 months vs. 2.8 months; hr: 0.44; *p* < 0.01)^7^. For poor-risk patients, the agent temsirolimus (an inhibitor of the mammalian target of rapamycin) has similarly led to an improvement in pfs; moreover, temsirolimus is the first of the targeted therapies for mrcc to also improve os (7.3 months in patients receiving interferon alfa vs. 10.9 months in patients receiving temsirolimus alone; hr: 0.73; *p* = 0.008)^8^.

Beyond small-molecule tkis, monoclonal antibody (mAb) therapy for mrcc has been evaluated in other recent studies. Bevacizumab, a mAb with specificity for vegfr, was assessed in a phase iiitrial comparing the combination of bevacizumab and interferon alfa with interferon alfa and placebo. In 649 randomized patients, a significant improvement in response rate was observed with the addition of bevacizumab (13% vs. 31%; *p* < 0.0001), with most of responses being characterized as partial. Median pfs was improved to 10.2 months from 5.4 months with the addition of bevacizumab (hr: 0.63; *p* < 0.0001), and at the time of this (interim) analysis, median os in the bevacizumab-containing arm had not been reached 9.

### 2.2 Rationale for the Combination of Bevacizumab and Imatinib

The combination of bevacizumab and imatinib exclusively has not been reported in the existing literature, but a recent trial tested these agents in combination with erlotinib. That study built on data from a randomized phase ii trial of first-line treatment of mrcc with bevacizumab and erlotinib as compared with bevacizumab and placebo. In 104 patients, the combination of bevacizumab and erlotinib did not significantly improve pfs (9.9 months vs. 8.5 months; hr: 0.86; *p* = 0.58). Interestingly, median os was not reached in the bevacizumab monotherapy group, but a median os of 20 months was noted with bevacizumab and erlotinib^10^.

A separate phase i/ii trial that assessed bevacizumab and erlotinib in combination with imatinib demonstrated a median pfs of 8.9 months and a median os of 20 months, thus mirroring the data obtained for single-agent therapy with bevacizumab. However, the addition of imatinib led to a marked increase in reported grades 3 and 4 toxicities, including diarrhea, rash, and fatigue 11. Thus, although the combination of erlotinib and bevacizumab is well tolerated, efficacy data do not seem to support the addition of the erlotinib. Furthermore, addition of imatinib to this regimen appears to generate unacceptable toxicity.

The case described the present report represents a signal of activity for the combination of bevacizumab and imatinib exclusively. To date, no trials for this specific combination of agents are being conducted, although there are theoretical merits for the combination. Imatinib is an inhibitor of multiple tyrosine kinases, including Abl, Bcr–Abl, pdgfr, and c-Kit^1^. Among these targets, pdgfr is of particular relevance to rcc and is a demonstrated target of sunitinib (together with vegfr and Flt-3)^5^.

A retrospective analysis of 64 patients undergoing partial or radical nephrectomy for rcc suggested that immunohistochemical staining for pdgfr is a statistically significant prognostic factor for os on multivariate analysis 12. Array studies of tissue suggest that rcc overproduces pdgfd, a recently discovered member of the pdgf family. Transfection of pdgfd in human rcc cell lines implanted in mice with severe combined immunodeficiency led to increased cellular proliferation and migration. Inhibition of pdgfr β activity by imatinib led to a decline in tumour growth, suggesting a therapeutic role of imatinib in rcc 13.

Imatinib may target the kinase domain of cell surface receptors, but bevacizumab acts in a unique manner—through specific binding to vegf. *In vitro* and *in vivo* assays have both demonstrated the role of vegf as a mitogen and angiogenesis inducer; bevacizumab has been shown in experimental models to reduce the growth rate of tumours, with decreased vessel density in bevacizumab-treated specimens 14. Other studies further suggest that bevacizumab may play a role in tumour vessel normalization, thereby alleviating tumour hypoxia and allowing for improved drug delivery to tumour tissue 15. The latter hypothesis suggests a mechanism by which imatinib could potentially synergize with bevacizumab.

## 3. CONCLUSIONS

Results from the phase i/ii trial combining bevacizumab, erlotinib, and imatinib should not discourage further exploration of the combination of bevacizumab and imatinib exclusively. The excessive toxicity incurred with the addition of imatinib could be a result of overlapping downstream targets: although imatinib and erlotinib inactivate phosphorylation at distinct tkis, the result is inhibition of common mitogenic pathways such as the pi3k–Akt signalling cascade 16,17. Given the exceptional tolerance and prolonged stable disease observed in the present case report, clinical studies of bevacizumab and imatinib should be pursued.

## 4. RESEARCH SUPPORT AND DISCLAIMERS

No research funding was applied toward the effort reported here, and this research has not been previously presented.
